# Ultrasonography of the multifidus muscle in student circus artists with and without low back pain: a cross-sectional study

**DOI:** 10.1186/s13102-023-00661-z

**Published:** 2023-04-07

**Authors:** Bianca Rossini, Meagan Anstruther, Daniel Wolfe, Maryse Fortin

**Affiliations:** 1 Department of Health, Kinesiology and Applied Physiology, Concordia University, 7141 Sherbrooke Street W, SP-165.29, Montreal, QC H4B 1R6 Canada; 2 PERFORM Centre, Concordia University, Montreal, QC Canada; 3 Centre de Recherche Interdisciplinaire en Réadaptation (CRIR), Montreal, QC Canada

**Keywords:** Multifidus muscle, Ultrasound imaging, Low back pain, Cross-sectional area

## Abstract

**Background:**

Degenerative structural changes and functional deficits of the lumbar multifidus (LM) muscle were observed in athletes with low back pain. While spinal injuries are common in circus artists, there is no information on LM characteristics in this population. The aims of this study were to investigate LM morphology and function and explore the relationship between LM characteristics and low back pain in male and female circus artists.

**Methods:**

31 college circus students were recruited. Participants completed an online survey to acquire demographic data and low back pain history. Body composition was measured using multi-frequency bio-impedance analysis. Ultrasound examinations at the fifth lumbar vertebrae in prone and standing positions were performed to assess LM cross-sectional area, echo-intensity, thickness. Independent and dependent t-test assessed the difference between sex and side, respectively. The relationships between measures were assessed with Pearson’s correlations. The LM characteristics’ difference between artists with and without low back pain (group binary variable) was assessed with Analysis of covariance using lean body mass, height and % body fat as continuous covariates.

**Results:**

Males had significantly larger LM cross-sectional area, lower echo-intensity and greater thickness change from rest to contracted than females. LM cross-sectional area asymmetry in prone was greater in artists reporting low back pain in the previous 4-weeks (p = 0.029) and 3-months (p = 0.009). LM measures were correlated with lean body mass, height, and weight (r = 0.40–0.77, p ≤ 0.05).

**Conclusion:**

This study provided novel insights into LM characteristics in circus artists. Greater LM asymmetry was observed in artists with a history of low back pain. In accordance with previous studies in athletes, LM morphology and function were highly correlated with body composition measurements.

## Background

Pain and injury are detrimental to a circus artist’s health and career.[1, 2] Student circus artists push their limits daily when loading, twisting and bending their vertebral column to achieve greater range.[1, 3–5] Spinal injuries are reported as the second most injured body part in circus artists and the lumbar spine as the most affected spinal section in the sparse research on circus injuries.[5–7] A study with an Australian circus school reported that 14% of all injuries were to the lumbar spine and that such injuries required the most initial and follow-up treatment.[5] Identifying the LBP profile of circus artists may lead to a more sustainable practice, decrease the risk of spinal injuries and assist with the screening of overuse injuries to the vertebral column.[5].

The lumbar multifidus muscle (LM) provides segmental stabilization and proprioception to the lumbar spine,[8] and it is well-recognized that LM morphology differs between male and female athletes; male athletes have a larger LM and greater percent thickness change as compared to female athletes. [9, 11] Smaller LM and greater asymmetry between sides has also been linked to LBP in some athletic [9–12] and non-athletic populations.[13, 14] Ultrasonography is a common and reliable method to assess LM size, echo intensity and percent thickness change from a rested to contracted state.[15–18] To date, however, most studies assessing LM morphology were conducted with non-performing athletes, and only few artistic populations with demands similar to circus artists have been investigated. Professional ballet dancers with LBP were reported to have smaller LM size and elite gymnasts with sway-back posture have reduced LM contraction when compared to their asymptomatic counterparts.[12, 19] LM asymmetry was also observed in elite ballroom dancers, but it was not related to LBP.[20] While previous findings suggest that LM morphology may be unique to each artistic population and reflect sport specific demands, we are not aware of any previous studies that have assessed LM characteristics in circus artists.

As muscle morphology is influenced by age, sex, physical activity levels and body composition,[21, 22] adjusting for such anthropometric factors is critical when assessing the relation between LM morphology and LBP. Body mass index (BMI) is most frequently used to adjust for inter-subject variability; however, it remains a poor indicator of body composition, especially in athletic populations.[22] While dual energy X-ray absorptiometry is the gold standard to assess body composition, it is costly and not readily accessible. Multi-frequency Bioimpedance Analysis (MF-BIA) is an accurate, quick, non-invasive, portable and affordable alternative.[23].

Given the scarce research on circus artists, we investigated the relationship between LM characteristics and LBP in this unique population. The aims of this study were to 1) investigate LM morphology and function and their relations with body composition in male and female artists, and 2) to examine the relationship between LM characteristics and LBP status. We hypothesized that circus artists with LBP would have smaller LM, greater side-to-side asymmetry, and reduced percent thickness change.

## Methods

### Participants

31 students aged 21.06 ± 2.56 pursuing a three-year diploma of collegial studies in circus arts were recruited from the National Circus School (n = 25) and the Quebec Circus Arts School (n = 6). Exclusion criteria were any history of spinal fracture, spinal surgery, or visible spinal deformities (i.e., scoliosis > 10°). All data was collected during a high intensity-training period in Fall 2021 semester. Due to Covid-19, the training hours of the 2020–2021 school year had been cut down by 50%, and artists had a reduced training schedule (e.g., training an average of two to three hours/day). In Fall 2021 during data collection for the current study, training hours were increased to five to six hours/day (75%). Informed consent was obtained from all participants. All methods were carried out in accordance with relevant guidelines and regulations. The study was approved by Concordia Ethics (CER: 30,014,948) and by National Circus School Ethics Committee (CER 2122-07C).

### Procedures

Participants completed a self-reported online survey on demographics, training, injury history and LBP. Injury was defined as any injury requiring medical attention. Participants were asked to answer “yes” or “no” to the presence of LBP in the past 4-weeks and 3-months prior the ultrasound assessment. If participants answered “yes” to the presence of LBP, they were asked to specify pain intensity using a numerical pain rating scale (0 = no pain; 10 = worst possible pain), pain location (left, right, centered) and pain duration in weeks. In accordance with previous related studies,[9, 11, 33, 34] separate analyses were conducted for the presence of LBP at 4-weeks and 3-months prior (refer to statistical analysis). Participants with a history of LBP also completed the Oswestry Disability Index (ODI)[24], the Athlete Disability Index (ADI),[25, 26] and the Pain Catastrophizing Scale (PCS)[27] questionnaires.

### Body composition

Height was recorded with a stadiometer (Doran Scales, DS5100). The measure was taken in the morning whenever possible to have minimal interference with the students’ training schedule. Participants were also instructed to fast for two hours, drink minimal amounts of water and not exercise prior to the body composition measurement.[28] Most students complied to this protocol, however, nine students ate and two students trained prior to acquiring body composition measurements. During the assessment, participants wore minimal clothing and removed all metal and footwear. Due to accessibility and transportability of the devices between study sites, a MF-BIA Inbody 230 (InBody CANADA, Ottawa, ON, CA) was used at the Montreal site (n = 25) and an Impedimed SFB7 (ImpediMed Inc. Carlsbad, CA, USA) was used in Quebec City (n = 6). Body composition measurements were obtained strictly following the equipment respective instructions and exported in a spreadsheet.

### Ultrasound Imaging

LM was assessed using a LOGIC e ultrasound (GE Healthcare, Milwaukee, WI) with a 5-MHz curvilinear probe. The following imaging parameters were used: 5 MHz frequency, 60 gain, 8.0 cm depth.[29] Reliability and validity of ultrasound imaging for LM size and thickness has been established.[16, 18, 29] The examiner was blinded to the clinical symptoms of the participants.

#### LM measurements

Participants lay prone on a therapy table with a pillow under their abdomen to flatten the lumbar curve,[15, 29] and with their arms relaxed on each side at the shoulder level while relaxing the lumbar musculature. The spinous process of L5 was located by palpation and confirmed visually on the ultrasound. Gel was applied to the skin and the ultrasound probe was placed transversely over the spinous process of L5. Three transverse images were taken bilaterally to obtain LM CSA. For participants with larger LM, the left and right muscle sides were imaged separately.

**Fig. 1:**
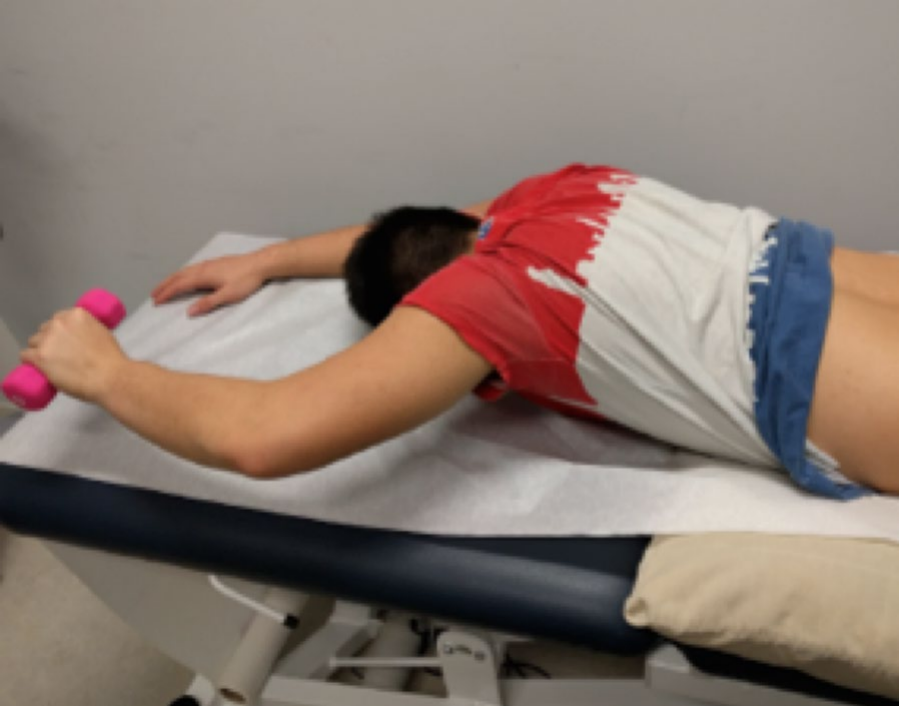
Position to acquire LM thickness measurement during contraction via contralateral arm lift

LM thickness measurements were acquired in a parasagittal view. Three images were taken at rest and three images during contraction. Participants held a handheld weight overhead with a 90° flexion in the elbow based on their bodyweight (< 68.2 kg = 0.68 kg weight, 68.2–90.9 kg = 0.9 kg weight, > 90.9 kg = 1.36 kg weight) [15] and performed a contralateral arm lift by lifting the weight 5 cm off the table for 3 s to obtain an image during contraction as shown in Fig. 1. Participants had 1 practice trial followed by 3 recorded arm lifts on each side.

All LM measurements were repeated in a standing position. Participants stood barefoot on the ground with their arms relaxed on each side. They were instructed to march on the spot and stop where their feet land in their normal upright resting position. The same procedure as described above was used was used to acquire LM CSA and thickness at rest. For LM thickness measurements during contraction, a contralateral arm lift was performed with the shoulder in 90° flexion and an elbow extension with the palm facing down.[30].

### Image assessment

**Fig. 2:**
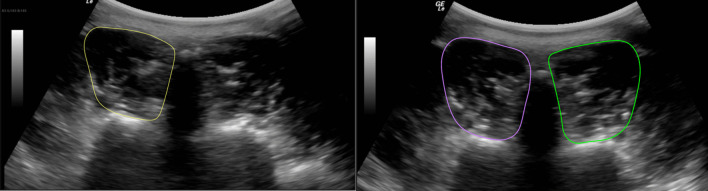
Transverse image of the lumbar multifidus muscle at L5 showing the cross-sectional area (CSA), in prone (left) and in standing (right) positions

**Fig. 3:**
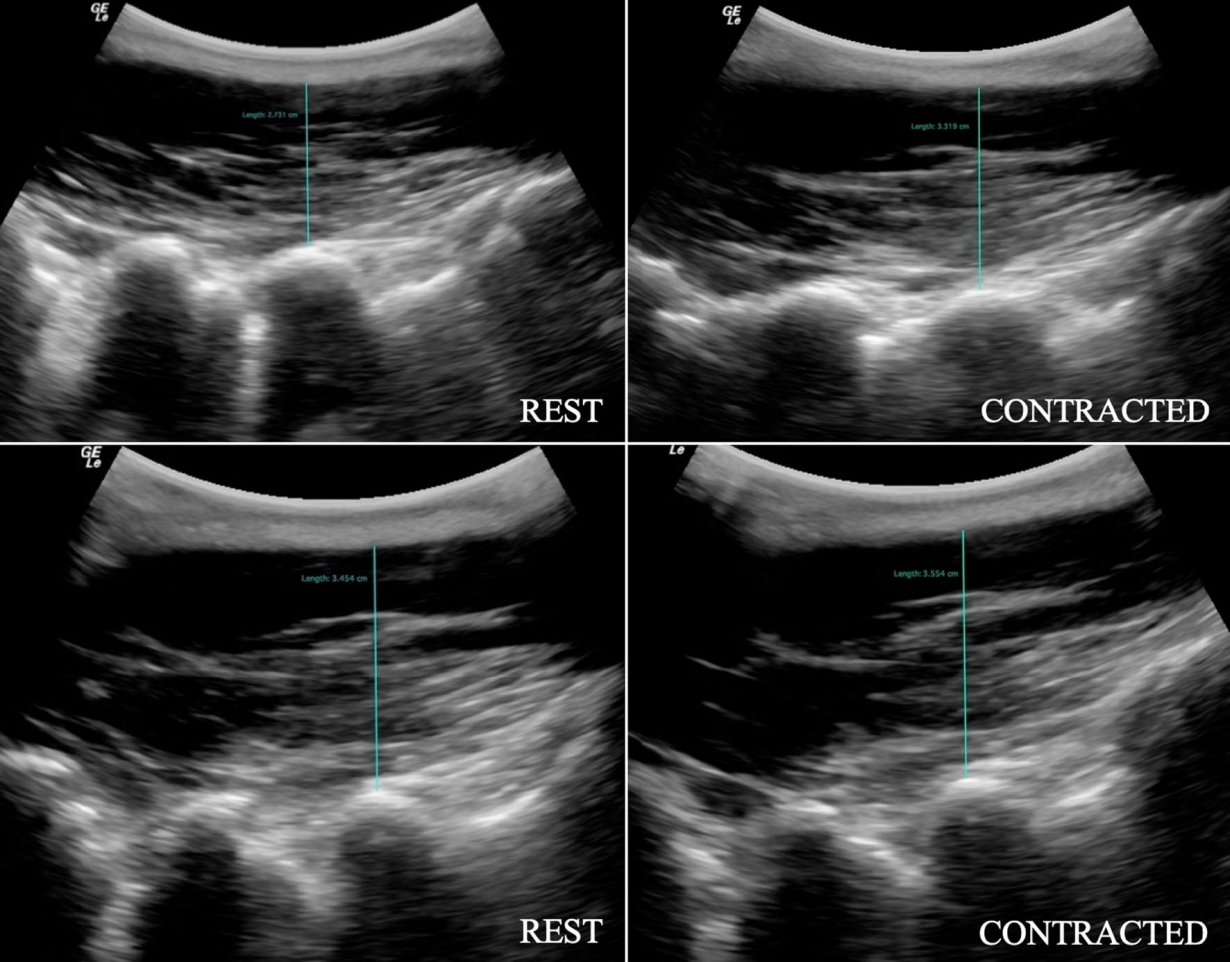
Parasagittal image of the lumbar multifidus muscle at L5 illustrating LM thickness at rest and during contraction, in prone (top row) and standing (bottom row) positions

Ultrasound images were stored and analyzed offline. LM CSA was measured by tracing the muscle borders manually (Fig. 2). LM CSA asymmetry was calculated using the following formula: % asymmetry = [(larger side – smaller side)/larger side × 100]. LM function was assessed by tracing the muscle thickness (Fig. 3) and calculating the % thickness change as follows: thickness % change = [(thickness contraction – thickness rest)/thickness rest) × 100].[9] Echo intensity (EI) values were measured using a gray scale analysis and standard histogram function via the Horos DICOM viewer software (version 4.0.0 RC5). Higher EI values reflect greater amounts of intramuscular fat and connective tissue.[29, 31] All measurements were acquired on three different images, and the average was used in the analysis. One participant was recovering from a shoulder injury and unable to perform the right contracted thickness measure in prone; only the three images from contraction on the right side in prone were excluded from the analysis for this participant. From the 372 images, eight additional individual images were discarded due to lack of clarity.

#### Reliability

The reliability of the examiner to acquire the ultrasound measurements was assessed prior to this study, following a familiarization and training period with the equipment*.* Within-day intra-rater reliability (n = 10) was excellent for all measurements with intraclass correlation coefficient (ICC) ranging from 0.91–0.99.

### Statistical analysis

Descriptive tables for participants’ characteristics, LBP status and LM measures were generated. Paired t-tests were used to compare LM size and function between the left and right sides, with the exception of LM CSA asymmetry for females, where the Wilcoxon signed-rank test was used as this measure was not normally distributed. Comparisons between sexes were assessed using unpaired t-tests, except for LM CSA asymmetry where the Mann–Whitney U test was used as this measure was not normally distributed in females. Pearson’s correlations were used to assess the relationship between body composition and LM characteristics. Analyses of covariance (ANCOVA) were used to assess the difference in LM characteristics between artists with and without LBP, and separate analysis were conducted for the presence of LBP at 4-weeks and 3-months prior to data collection. Height, % body fat and lean body mass were used as covariates. All tests were performed using SPSS (version 26.0.0.0) with significance level set at < 0.05.

## Results

The artists’ demographic and LBP characteristics are presented in Table 1 and 2. The average years of circus training was 7.97 ± 4.26 years. 18 students (58.1%) reported the presence LBP.

**Table 1:** Participants' Characteristics ((mean + SD) or n)

	All (n = 31)	Male (n = 13)	Female (n = 18)
Age (yrs.)	21.06 ± 2.56	21.46 ± 2.30	20.78 ± 2.76
Height (cm)	168.39 ± 8.63	175.12 ± 5.72	163.53 ± 6.98
Weight (kg)	63.37 ± 8.67	68.77 ± 8.14	59.48 ± 6.90
Total lean mass (kg)	54.06 ± 9.57	61.77 ± 8.31	48.50 ± 5.91
Total body fat %	14.94 ± 5.64	10.22 ± 4.40	18.35 ± 3.63
Body mass index	22.28 ± 2.09	22.39 ± 2.28	22.20 ± 2.02
Program Year (n)			
Preparatory year	7	3	4
First year	7	1	6
Second year	9	4	5
Third year	8	5	3
Years of circus training (yrs.)	7.97 ± 4.26	9.85 ± 4.02	6.61 ± 4.00
Type of artists (n)			
Specialists	24	7	17
Generalists	7	6	1
Main Discipline (n)			
Floor Acrobatics	11	8	3
Aerial Acrobatics	14	2	12
Balancing	5	3	2
Juggling	1	0	1
Time training main discipline (yrs.)	4.74 ± 2.58	5.46 ± 1.98	4.22 ± 2.88
Time training main discipline (Hr / week)	7.94 ± 2.78	8.15 ± 4.00	7.78 ± 1.52
Hand Grip Preference (n)			
Left	3	1	2
Right	22	8	14
No Preference	6	4	2
Medical History in previous 12 months (n)			
Students who reported injuries	22	10	12
Injuries to the head, neck, trunk	8	1	7
Injuries to the arms	12	7	5
Injuries to the legs	10	3	7

**Table 2:** LBP Characteristics ((mean + SD) or n)

	All (n = 18)	Male (n = 7)	Female (n = 11)
Total LBP reports	18	7	11
in previous 4 weeks (answered “yes”)	14	6	8
in previous 3 months (answered “yes”)	16	6	10
LBP in previous 4 weeks			
Duration (weeks)	2.35 ± 0.44	1.48 ± 1.36	3.01 ± 1.57
Location (n)			
Centered	7	4	3
Left	3	0	3
Right	4	2	2
Intensity (0–10 scale)	4.36 ± 2.21	4.25 ± 1.13	4.44 ± 2.85
LBP in previous 3 months			
Duration (weeks)	17.65 ± 8.82	6.00 ± 6.06	24.00 ± 44.42
Location (n)			
Centered	10	5	5
Left	3	0	3
Right	3	1	2
Intensity (0–10 scale)	4.63 ± 2.29	4.50 ± 1.67	4.70 ± 2.68
Questionnaires on LBP			
ODI scores %	9.33 ± 7.67	9.14 ± 6.82	9.45 ± 8.49
ADI scores %	16.20 ± 11.36	14.29 ± 7.25	17.42 ± 13.56
PCS score (/52)	10.63 ± 8.23	9.00 ± 7.70	11.58 ± 8.70
ODI interpretation results, No			
Minimal disability	17	7	10
Moderate disability	1	0	1
Severe disability	0	0	0
ADI interpretation results, No			
Minimal disability	12	5	7
Moderate disability	5	2	3
Severe disability	1	0	1

### LM characteristics

LM characteristics for males (n = 13) and females (n = 18) are presented in Table 3. In prone, the right LM CSA was significantly greater in males (p < 0.01). EI was significantly greater in females (p < 0.01), and larger on the right side within females (p = 0.01). LM thickness at rest (left side), contracted (both sides), and % thickness change (right side) were significantly larger in males (p < 0.05). There was no difference in LM CSA asymmetry between sexes.

**Table 3:** LM characteristics in circus artists

	Female (n = 18)		Male (n = 13)	
	Right	Left	Right	Left
Prone				
CSA (cm^2^)	**6.83 ± 1.13**	6.86 ± 1.11	**8.01 ± 1.19**	7.88 ± 1.10
CSA asymmetry (%)	2.91 ± 3.89 ^†^		2.21 ± 2.33 ^‡^	
Echo intensity (arbitrary units	51.76 ± 10.74*	**47.02 ± 13.13**	**37.23 ± 7.11**	**36.40 ± 5.95**
Thickness (cm)				
*Rest*	2.65 ± 0.26	**2.70 ± 0.27**	2.87 ± 0.39	**2.96 ± 0.31**
*Contracted*	**2.80 ± 0.27**	**2.87 ± 0.28**	**3.20 ± 0.43**	**3.24 ± 0.31**
*Percentage change (%)*	**5.71 ± 5.04**	6.46 ± 4.20	**11.78 ± 4.71**	9.52 ± 5.31
Standing				
CSA (cm^2^)	**7.70 ± 1.23**	**7.79 ± 1.16**	**9.44 ± 1.25**	**9.48 ± 1.14**
CSA asymmetry (%)	2.17 ± 2.42 ^†^		3.07 ± 2.91 ^‡^	
Thickness (cm)				
*Rest*	3.06 ± 0.35*	**3.19 ± 0.30**	**3.45 ± 0.45**	**3.52 ± 0.39**
*Contracted*	**3.25 ± 0.34**	**3.31 ± 0.30**	**3.57 ± 0.43**	**3.66 ± 0.36**
*Percentage change (%)*	6.47 ± 5.39	3.99 ± 2.57	3.61 ± 2.78	4.10 ± 2.73

^*^Indicates difference (p < 0.05) between left and right within sex

^†^Indicates a Wilcoxon signed-rank test was used to compare measure within same sex

^‡^Indicates a Mann–Whitney U test was used to compare a measure between sexes

Bold indicates difference (p < 0.05) between sex

In standing, LM CSA, thickness at rest and contracted on both sides were larger in males (p < 0.05). Female artists had greater right LM thickness at rest (p = 0.036). There was no difference in LM CSA asymmetry between sexes.

### Associations between LM characteristics and body composition

LM CSA was significantly correlated with height (prone: r = 0.55, p < 0.01; standing: r = 0.66, p < 0.001), weight (prone: r = 0.73, p < 0.001; standing: r = 0.74, p < 0.001), total lean mass (prone: r = 0.73, p < 0.001; standing: r = 0.77, p < 0.001) and % body fat (prone: r = -0.43, p = 0.02; standing: r = -0.51, p < 0.01). LM thickness at rest and contraction in prone and standing had similar significant correlations. LM EI was only correlated to % body fat (r = 0.48, p = 0.01). The EI was significantly and negatively correlated with percent thickness change in prone (r = -0.40, p = 0.03); however, this correlation was not significant in standing (p = 0.24). LM CSA (prone: r = -0.43, p = 0.02; standing: r = -0.51, p < 0.01), contracted thickness in prone (r = -0.43, p = 0.02) and thickness measures in standing (rest: r = -0.37, p = 0.04; contracted: r = -0.36, p < 0.05) were correlated with total % body fat. BMI was significantly correlated with CSA in prone (r = 0.44, p = 0.01); however, BMI was not significantly correlated with CSA in standing (r = 0.35, p = 0.06). All thickness measures in prone and standing were significantly correlated with BMI (r = 0.50–0.57, p < 0.01). The correlation coefficients for BMI were smaller than other body composition measures.

### LBP comparisons

Comparisons of LM characteristics between artists with and without LBP in the past 4- weeks and 3-months are presented in Table 4 and 5, respectively. LM asymmetry in prone was significantly greater in artists reporting LBP in the previous 4-weeks (p = 0.022, η^2^ = 0.180) or 3-months (p = 0.010, η^2^ = 0.224). There were no other significant differences in LM characteristics between artists with and without LBP.

**Table 4:** Adjusted means^a^ (mean (SE)) of LM measurements in prone and standing for artists with and without LBP in past 4-weeks

			p-values	η^2^
	No LBP (n = 17)	LBP (n = 14)		
Prone				
CSA (cm^2^)^*b*^	*7.25 (0.23)*	*7.38 (0.25)*	*0.713*	***0.005***
CSA asymmetry (%) ^*b*^	**1.32** **(0.79)**	**4.19 (0.88)**	**0.029**	**0.171**
Echo intensity (arbitrary units) ^*b*^	45.65 (2.69)	42.26 (2.99)	0.431	**0.024**
Thickness (cm)				
*Rest*	2.76 (0.06)	2.79 (0.06)	0.780	**0.003**
*Rest * *Asymmetry (%)*	6.28 (1.25)	5.77 (1.40)	0.797	**0.002**
*Contracted *^*b*^	3.00 (0.07)	3.01 (0.07)	0.910	**0.001**
*Contracted Asymmetry (%)*	7.79 (1.18)	6.80 (1.27)	0.593	**0.011**
*Percentage change (%)*	8.55 (1.14)	8.04 (1.23)	0.773	**0.003**
*Percentage change Asymmetry (%)*	3.57 (0.88)	5.17 (0.95)	0.251	**0.050**
Standing				
CSA (cm^2^) ^*b*^	8.38 (0.25)	8.56 (0.28)	0.664	**0.007**
CSA asymmetry (%) ^*b*^	2.58 (0.69)	2.51 (0.77)	0.948	**0.000**
Thickness (cm)	3.28 (0.07)	3.28 (0.08)		
*Rest *^*b*^			0.994	**0.000**
*Rest Asymmetry (%)*	5.66 (1.27)	5.93 (1.42)	0.894	**0.001**
*Contracted*^*b*^	3.42 (0.07)	3.43 (0.08)	0.944	**0.000**
*Contracted Asymmetry (%)*	6.14 (1.04)	4.43 (1.20)	0.314	**0.039**
*Percentage change (%)*	4.78 (0.72)	4.55 (0.84)	0.845	**0.001**
*Percentage change * *Asymmetry (%)*	2.42 (0.83)	3.90 (0.96)	0.279	**0.045**

^a^Adjusted means for lean body mass and height

^b^Adjusted means for lean body mass, height and total body fat %

bold = P < 0.05

**Table 5:** Adjusted means^a^ (mean (SE)) of LM measurements in prone and standing for artists with and without LBP in past 3-months

			p-values	η^2^
	No LBP (n = 15)	LBP (n = 16)		
Prone				
CSA (cm^2^) ^*b*^	7.27 (0.25)	7.35 (0.24)	0.822	**0.002**
CSA asymmetry (%) ^*b*^	**0.82 (0.83)**	**4.30 (0.80)**	**0.009**	**0.232**
Echo intensity (arbitrary units) ^*b*^	44.62 (2.99)	43.65 (2.88)	0.830	**0.002**
Thickness (cm)				
*Rest*	2.75 (0.06)	2.80 (0.06)	0.544	**0.014**
*Rest Asymmetry (%)*	7.55 (1.30)	4.64 (1.25)	0.137	**0.080**
*Contracted *^*b*^	3.00 (0.07)	3.01 (0.07)	0.940	**0.000**
*Contracted Asymmetry (%)*	7.96 (1.22)	6.69 (1.22)	0.493	**0.018**
*Percentage change (%)*	8.68 (1.19)	7.95 (1.19)	0.684	**0.006**
*Percentage change Asymmetry (%)*	4.46 (0.95)	4.18 (0.95)	0.845	**0.001**
Standing				
CSA (cm^2^) ^*b*^	8.56 (0.28)	8.37 (0.27)	0.638	**.009**
CSA asymmetry (%) ^*b*^	2.67 (0.76)	2.43 (0.73)	0.831	**0.002**
Thickness (cm)	3.28 (0.08)	3.27 (0.08)		
*Rest *^*b*^			0.934	**0.000**
*Rest Asymmetry (%)*	4.90 (1.35)	6.60 (1.31)	0.398	**0.027**
*Contracted *^*b*^	3.42 (0.08)	3.43 (0.07)	0.946	**0.000**
*Contracted Asymmetry (%)*	5.78 (1.13)	5.04 (1.13)	0.675	**0.007**
*Percentage change (%)*	4.65 (0.78)	4.71 (0.78)	0.957	**0.000**
*Percentage change Asymmetry (%)*	3.18 (0.91)	2.94 (0.91)	0.863	**0.001**

^a^Adjusted means for lean body mass and height

^b^Adjusted means for lean body mass, height and total body fat %

bold = P < 0.05

## Discussion

Few studies have assessed LM characteristics in artistic and athletic populations with LBP. We are not aware of any studies assessing LBP profile and LM measures in circus artists. This study provides novel insights with regards to LM morphology and function in male and female circus artists, with and without a LBP history. Overall, 18 artists (58%) reported LBP, out of which 45% and 52% reported LBP in the previous 4-weeks and 3-months, respectively. Other studies investigating performing arts reported higher LBP prevalence, with 74% of professional ballet dancers reporting chronic LBP and 63% of ballroom dancers experiencing LBP from months to years.[19, 20].

Our findings revealed that LM CSA in circus artists was comparable to elite dancers [19, 20] and greater than non-athletic healthy subjects of similar age and higher % body fat.[32] Other studies in athletic populations with larger stature (e.g., hockey, rugby, American football, soccer) reported greater LM CSA at the same level.[9–11, 33] LM morphology varies in different athletic populations as each sport requires different physicality and physical profile. For example, the right/dominant LM CSA was reported to be larger in all ballet dancers which was attributed to the dominance and lateral training bias.[19] Similar lateral bias results were reported in elite ballroom dancers and in varsity football players, which had a larger LM CSA on their dominant side due to the nature of the art or sport.[20, 34] In contrast, circus training favors symmetrical training, especially circus schools. This could partly explain the very small asymmetry values observed in our sample. Furthermore, circus artists tend to be very lean with small stature and have unique demands to stabilize their vertebral column without having to react to unexpected hits or loss of stability such as in football or hockey.[9–11, 33, 34] In accordance with previous studies,[9, 11, 33, 34] we also observed significant difference in LM morphology characteristics between male and female circus artists. Our sample of males were taller, heavier, and had lower percent body fat then the females, which likely explains the larger CSA, lower EI and larger thickness measures prior to adjusting for anthropometric differences.

Few studies have examined LM morphology and function in standing.[9, 11, 33, 34] Assessing LM characteristics in a functional upright position may result in better implications for performance and injury prevention.[9] LM CSA is expected to increase from a prone to standing position as the muscle contracts in an active and supportive role. On the contrary, thickness change is greater in prone than standing, as the LM muscle is already contracted in a stabilizing role while standing.[29, 35] Our results corroborate with previous studies in athletes and showed greater LM CSA and smaller thickness change in standing,[9, 11, 33, 34] except for the right side in female artists where larger thickness change was observed in standing. This could be an adaptation related to the demands of the art; however, another explanation revolves around the choice of handheld weight. Most female artists weighted less than 150 pounds, hence, used the smallest handheld weight (e.g., 0.68 kg).[15] Indeed, the original study that determined the required handheld weight to elicit a 20–30% LM involuntary contraction was developed in a general non-athletic population.[15] Circus artists are leaner and have stronger and more flexible shoulders than the general population.[36] While this protocol has been used for other athletic and artistic populations such as rhythmic gymnastics,[9, 11, 12, 33, 34] it is possible the smallest handheld weight was too small to elicit the expected involuntary LM contraction in circus artists due to their increased shoulder control, strength and flexibility. Additional aspects of LM neuromuscular control should be investigated in circus artists.

In accordance with previous studies in varsity athletes,[9, 11, 33, 34] LM CSA was positively correlated with lean body mass, weight, and height; these correlations were stronger in circus artists. LM CSA was negatively correlated with % body fat as was reported in soccer players (r =  − 0.41),[9] contrary to other varsity athletes where it was positively correlated.[11, 33, 34] Percent body fat was positively associated with EI as in varsity athletes (r = 0.76),[11, 34] and negatively associated with LM CSA as in varsity soccer players.[9] The negative correlation between total percent body fat and thickness change in prone was also reported in football athletes.[34] This finding is in accordance with Schryver et al. and provide additional evidence that body composition may negatively affect muscle function.[34] Contrary to studies in varsity athletes,[9, 11, 33] BMI was correlated with LM CSA in our sample. In comparison to previous study with athletes of bigger statures, our sample of circus artists had lower percent body fat and large developed muscles.[9, 11, 33, 34, 37] Of note, lean body mass was the best predictor of LM CSA in circus athletes. Thus, body composition predictors for LM size may vary between populations.

Contrary to our hypothesis, circus artists with a history of LBP did not have smaller LM. This finding was also reported in ballroom dancers and other elite athletic populations.[20, 38, 39] However, professional ballet dancers with hip or back pain had a smaller LM than dancers without pain at the lower lumbar levels (L3 to L5).[15] These inconsistent findings may relate to the specificity of each art. LM EI in circus artists was not associated with LBP status but highly correlated with body composition. While we are not aware of any previous studies that investigated LM EI in performing arts, this findings corroborates with previous studies in varsity athletes.[9, 11, 19, 20] Furthermore, we found no significant differences in LM thickness change between artists with and without LBP. While elite female artistic gymnasts with sway back posture had a decrease in thickness,[13] literature findings with regards to LM dysfunction in athletic populations with LBP are mixed.[9, 33].

Our results, however, revealed that circus artists with LBP had greater CSA asymmetry in prone, a finding also reported in professional ballet dancers.[19] Previous studies in non-athletic populations suggested side-to-side asymmetry above 8–10% as a probable threshold related to pathology and LBP.[29, 32] However, circus artists rarely fall within the normative data due to the unique demands of circus. Despite a low-level asymmetry in our sample, the association between LBP and LM asymmetry suggest that a lower threshold value (below 8%) may be problematic and possibly led to LM dysfunction in circus artists. As this was a cross-sectional study, whether LM asymmetry happened prior to pain onset or as result of pain remains unclear. Furthermore, in accordance with ballet dancers, smaller LM was not associated with the side of pain identified.[15] Further investigations are required to confirm and expand our findings and determine if LM asymmetry could be an indicator or predictor of LBP in circus artists.

Limitations of this study include the small sample size which may have effected some analyses; however, it was comparable to other studies in elite athletes and professional performing artists.[9, 11, 19, 20, 33] The decreased number of training hours due to COVID and may have affected our overall results. While a few artists did not fully comply with the body composition protocol, this did not have a major impact on our measurements. We ran sub-analyses to compare the LM EI measures and pain levels between the 9 participants that did not comply with the body composition protocol and the rest of the group and found no difference in LM characteristics or pain outcomes. LM measurements were only obtained at a single spinal level. Further investigations in circus should examine LM characteristics at other spinal levels and other trunk muscles that contribute to segmental control and spinal stability.

## Conclusion

Student circus artists presented differences in LM morphology between males and females in prone and standing. Artists with LBP had larger side-to-side asymmetry in the LM CSA at fifth lumbar vertebrae imaged in prone. Our results suggest the importance to evaluate muscle characteristics at rest as well as in movement. Future research should explore clinically significant asymmetry threshold specific to circus and evaluate the effects of LM exercise intervention targeting LM muscle on reducing and preventing LBP in athletic and artistic populations.

## Data Availability

The datasets generated during and analyzed during the current study are available from the corresponding author on reasonable request.

## Abbreviations

LBP: Low back pain
LM: Lumbar multifidus
CSA: Cross sectional area
BMI: Body mass index
EI: Echo-intensity
ODI: Oswestry disability index
ADI: Athlete disability index
PCS: Pain catastrophizing scale
MF-BIA: Multi-frequency bio-impedance analysis
